# Standard setting for dental knowledge tests: reproducibility of the modified Angoff and Ebel method across judges

**DOI:** 10.1186/s12909-025-07822-3

**Published:** 2025-10-15

**Authors:** Ting Khee Ho, Noor Lide Abu Kassim, Lucy O’Malley, Reza Vahid Roudsari

**Affiliations:** 1 Division of Dentistry, School of Medical Sciences, Faculty of Biology, Medicine and Health, Manchester Academic Health Science Centre, University of Manchester, Oxford Road, Manchester, M13 9PL UK; 2 Department of Restorative Dentistry, Faculty of Dentistry, Universiti Kebangsaan Malaysia, Jalan Raja Muda Abdul Aziz, Kuala Lumpur, 50300 Malaysia; 3 Kulliyyah of Education, International Islamic University Malaysia, Jalan Sungai Pusu, Gombak, Selangor 53100 Malaysia

**Keywords:** Dental education, Education measurement, Standard setting, Angoff, Ebel, Passing score, Passing mark, Reproducibility of results, Malaysia

## Abstract

**Introduction:**

Criterion-referenced standard setting methods establish passing scores based on predefined competency levels. The credibility of these scores must be supported by validity evidence. This study evaluated the reproducibility of modified Angoff and Ebel standards across different test formats and panels in dental assessments. Inter-rater reliability for each method was also assessed.

**Methods:**

Twelve judges, selected via purposive sampling, were divided into two equal groups representing various specialisms. Each panel applied modified Angoff and Ebel methods to set standards for one-best answer (OBA) and short answer question (SAQ) items. Method replicability across panels was assessed using the Mann–Whitney U-test to compare passing scores between Groups A and B. The Wilcoxon signed-rank test compared passing scores between modified Angoff and Ebel within groups. Inter-rater reliability was estimated using the intraclass correlation coefficient for modified Angoff and Fleiss’ kappa for Ebel. Statistical analysis was conducted using IBM SPSS, with significance set at *p* < 0.05.

**Results:**

The median (IQR) years of teaching experience were 14.0 (17.0) for Group A judges and 21.5 (18.0) for Group B judges. In Group A, median (IQR) passing scores using modified Angoff were 49.75 (3.31) for OBA and 51.75 (6.13) for SAQ, with statistical no significant differences (*p* > 0.05) from Ebel OBA 47.38 (2.02), SAQ 49.50 (5.38). In Group B, modified Angoff passing scores were significantly higher than Ebel (*p* < 0.05): modified Angoff OBA 66.12 (3.31), SAQ 58.00 (7.50); Ebel OBA 55.92 (2.73), SAQ 49.50 (8.25). Passing scores were consistent across panels for SAQ but not for OBA. Inter-rater agreement, intraclass correlation coefficients (ICC) and Fleiss’ kappa were higher in Group A across both methods.

**Conclusion:**

Reproducibility of modified Angoff and Ebel standards across panels was mixed. Passing scores were consistent across judges for SAQ but varied for OBA in both methods. Group A showed consistency between modified Angoff and Ebel standards, whereas Group B had differing passing scores between both standards. These findings should be carefully considered when establishing defensible and reliable passing standards for dental knowledge assessments.

**Supplementary Information:**

The online version contains supplementary material available at 10.1186/s12909-025-07822-3.

## Background

Standard setting translates conceptual performance criteria into cutoff scores that distinguish between performance levels, such as pass-fail or proficiency categories (below basic, basic, proficient and advanced) [1, 2]. To ensure patient safety, dental institutions are accountable for assessing students against predefined standards in knowledge, skills and abilities to verify that they meet the minimum competency requirements set by professional regulatory bodies and adequately prepared for general practice [3–9].

In educational measurement, the passing scores are used to determine the eligibility of students to progress to the next academic year in dental courses or graduate. While traditionally the passing score may be set arbitrarily at fixed passing scores [10–13], such scores are often considered unfair because for ignoring candidates’ mastery of learning outcomes, test difficulty, individual abilities, or test objectives [14, 15].

Standard setting methods are broadly categorised as relative or absolute. Relative method ranks candidates’ scores, often forming a bell-shaped curve, with passing scores set at the mean minus a multiplier of the standard deviation, making judgements within a cohort [16, 17]. In contrast, the absolute method uses subject matter experts judging test items (test-centred standards) or candidates’ competence (examinee-centred standards) to establish passing scores that differentiate between competent and incompetent individuals [18–20]. Absolute standards are essential for maintaining the validity and accountability of certification and licensure examinations [21, 22].

The Nedelsky, Ebel and Angoff methods, including their variations, are test-centred absolute standards requiring subject matter experts (SMEs) to review individual test items and determine passing scores [2, 23–26]. These SMEs (also named panellists or judges provide judgement during this process [27]. A survey of 27 UK medical schools (79.4% response rate) found the Angoff method most commonly used in undergraduate finals for multiple-choice questions (MCQs) (57.5%), short answer questions (SAQs) (77.8%), and essays (50%). The Ebel method ranked second for MCQs (19.3%) and SAQs (11.1%) examined standard setting methods used in undergraduate final examinations [28].

In the Angoff method, judges estimate the probability that a ‘minimally competent examinee’ will answer each item correctly [26, 29]. This individual, also referred to as the ‘minimally acceptable person’, ‘minimally qualified candidate’, or ‘borderline candidate’, possesses the lowest level of competency deemed acceptable for the exam’s intended purpose [27]. Originally used with dichotomous scores with a single round of rating and modified versions introduced iteration rounds for consistency [30, 31], incorporated candidate performance data [32, 33] and extended to polytomous scoring formats, such as SAQs [34, 35].

In the Ebel method, judges first categorise items based on difficulty and content relevance within the test’s domains and intended learning outcomes, followed by estimating the probability that borderline candidates will answer correctly within each category [25]. The final passing score is calculated by multiplying the number of items in each category by the corresponding estimated probability, summing the products across all categories and dividing by the total number of items.

Validating pass–fail judgments made by standard setting panels requires procedural, internal, external and consequential validity evidence [1, 2, 20, 36–39]. Procedural validity involves the systematic implementation of standard setting procedures, including judge selection and training, data collection and analysis of the rating results, and process feedback [40–42]. Internal validity refers to the replicability of standard setting methods across judge panels and occasions, inter- and intra-rater agreement, and alignment with candidates’ performance data [20, 43]. External validity compares performance standards with alternative assessment methods, such as other standard setting approaches or employer feedback [44, 45]. Cizek describes validation of test scores as an ongoing process of collecting, summarising, and analysing evidence to determine the degree to which that evidence supports the intended interpretation of test scores produced by an instrument and the inferences drawn from them [46]. The validity of a standard setting procedure is crucial to ensure that the passing score as accurately reflects the performance standard based on the test’s intended purpose and minimum competency standard, as it impacts all stakeholders who rely on these for decisions [39].

The first comparison of passing standards among the Angoff, Ebel, National Board of Medical Examiners (NBME) and Guerin methods was conducted in the early 1980 s by the American National Board of Medical Examiners [47]. The NBME method used Rasch-based item difficulties, while the Guerin method combined item essentiality with difficulty for MCQs. The study found relatively consistent passing scores and standard errors for Angoff and Ebel compared to NBME and Guerin but did not detail sources of variance in standard-setting judgments. Swanson et al. (1990) addressed this to examine how iteration rounds and additional information affected passing standards with Angoff and Ebel variations. The study examined judges’ decisions across three rounds: independent ratings (Method I), feedback on initial ratings (Method II), and access to candidates’ performance data (Method III). Method II showed the highest inter-rater agreement and was preferred by judges [48]. Other studies suggest that providing judges with feedback and performance data before the final rating round enhances consistency, thereby improving reliability [33, 49–51]. Standard setting research has extended to the Korean Radiological Technologist Licensing Examination using Angoff, Ebel, Bookmark and Hofstee methods [52]. The Bookmark method uses item maps determined according to difficulty index extracted by item response theory (IRT) to place a cut score at the point where borderline candidates are expected to begin answering items incorrectly [53] while the Hofstee method combines expert judgment with an acceptable range of failure rates to set a compromised cut score [54]. Survey from judges indicated a preference for the Ebel method (57.1%), followed by Angoff method (28.6%) and Hofstee method (14.3%), with no support for Bookmark. Although the study examined reliability through correlations between methods, it did not analyse inter-rater agreement, an essential aspect of reliability [55]. The study ultimately recommended a consensus approach, adopting either the modified Angoff or Ebel method and verifying results with the Hofstee method for licencing examinations.

Although different standard setting methods yield varying passing standards, the primary focus should be on validity and practical implementation [42]. Iterative rounds, judge feedback and the incorporation of candidates’ performance data improve consistency among judges [30, 33, 56]. Studies have examined the reproducibility of the Angoff method across different judge panels [31, 57, 58]. Norcini and Shea (1992) found high consistency among four judge panels setting passing scores for two medical exams six months apart. A follow-up two years later, without candidate performance data, showed some variation in individual estimates, but the final average passing scores remained consistent with earlier results [58]. Studies in nonmedical fields also show Angoff-based methods produce consistent judgement and passing scores across panels [31, 57].

To date, no prior study has investigated the reproducibility of passing scores across different panels using Ebel method and limited research has assessed its reliability in estimating performance standards. Similarly, reproducibility of the modified Angoff and Ebel methods across different judges in dental assessments remained unexplored. This study aims to assess the reproducibility of passing scores within and across two groups using these methods in knowledge tests. The first objective is to compare passing scores between two independent panels (Groups A and B) using the modified Angoff and Ebel methods in the one-best answer (OBA) and SAQ formats. The null hypothesis posits no significant difference in the passing scores between the groups, irrespective of the standard setting method or test format. The second objective compares passing scores within each group between the two standard setting methods in OBA and SAQ formats, with the null hypothesis states no significant difference in passing scores between the two methods, regardless of groups or test format. The third objective evaluates inter-rater agreement across groups and standard setting methods to assess results reliability.

## Methods

### Setting and context

This study was conducted at a single centre at the National University of Malaysia (Universiti Kebangsaan Malaysia, UKM), a public dental university in Malaysia. The study employed an experimental design involving a three-day standard setting workshop. The workshop was led by the first author (TKH), a PhD candidate in dental education, in collaboration with a language educationist and psychometrician (AKNL) and a medical education specialist (MNAB). Both the psychometrician and the medical education specialist have extensive experience in conducting standard setting workshops. The first author, a lecturer at the UKM, had no administrative or teaching responsibilities during the research period.

### Assessment items

The undergraduate dental curriculum’s final-year coursework comprises five courses: Comprehensive Dental Care, Paediatric Dentistry, Orthodontics, Dental Public Health and the Undergraduate Research Project. The Comprehensive Dental Care (CDC) course integrates multiple disciplines, including endodontics, operative dentistry, oral medicine and pathology, oral and maxillofacial surgery, public dental health, prosthodontics and periodontics. This multidisciplinary approach enabled the recruitment of judges from diverse fields, ensuring both maximum variation and the minimum panel size required for the standard setting study. For this study, a theory paper from the CDC course was selected. It consisted of 20 MCQs in OBA format (each with four answer options) and 10 constructed-response questions in SAQ format. The SAQs were structured around clinical vignettes, each containing 2–4 sub-questions assessing clinical reasoning and case management. This theory paper accounted for 20% of the final-year CDC summative assessment and was previously administered in 2022 to a cohort of 38 students.

### Participants

The study participants served as judges. Inclusion criteria required participants to be dental specialists with at least three years of experience teaching final-year CDC students and the ability to fully commit to the workshop and standard setting activities. Visiting and part-time lecturers were excluded due to their limited involvement in the educational assessments.

### Recruitment

Judges were recruited from the dental faculty of UKM. Purposive sampling method used in this study aimed to achieve maximum variation among the participants. Faculty nominated a list of the lecturers teaching the CDC course, ensuring representation from each discipline. Invitations outlining the study’s purpose and workshop details were emailed to them. The initial target was 16 participants, allowing for a 20% dropout rate. Fourteen individuals expressed interest and written informed consent was obtained before participation. However, only 12 attended the workshop. These participants were purposively divided into two equal groups based on gender, ethnic, years of experience, dental discipline, and academic position.

### Ethical considerations

Study approval was approved by the ethics committees of the University of Manchester (2023-17408-31608) and the National University of Malaysia (UKM) (JEP-2023-204). During the proposal stage of the research, the dean of the dental faculty at UKM was consulted to discuss the plan for the standard setting workshop and meetings. In accordance with ethical guidelines, the workshop was scheduled during the semester break to avoid conflicts with participants’ regular academic commitments. Participants were reassured that their professional competency would not be assessed, and the data collected would have no impact on their career prospects. To maintain anonymity when submitting the ratings, judges were assigned random codes within their groups. Group A received single-digit codes (1–6), while Group B received two-digit codes (11–16).

### Procedure

A three-day workshop incorporating training was conducted. *Supplementary File 1* provides details of the workshop, including lecture topics, objectives, standard setting meetings and time allocation. Judges (participants) received training and applied standard setting methods. It was the first time that all participants had learned about standard setting using the modified Angoff and Ebel methods to familiarise themselves with these approaches. On the second day, they received a briefing on the standard setting procedures before being divided into two groups (Group A and Group B) in separate meeting rooms. They then applied the modified Angoff method to 20 OBAs and 10 SAQs, followed by the Ebel method. This manuscript presents findings relevant to the research objectives, while other results will be reported separately.

Group A was moderated by the lead author (TKH), and Group B by a psychometrician (AKNL). A medical education specialist (MNAB) served as an impartial observer to ensure standardisation and fairness. The detailed steps and instructions for standard setting meetings were separately discussed with the moderators before the workshop during preparation. These instructions were prepared in written point form alongside the timetable were provided for quick reference and moderators were reminded to adhere strictly to the procedures to maintain consistency across meetings.

Each method involved three rating rounds using answer-key-provided items. The first two rounds consisted of individual ratings, while the third round involved group discussion before the judges recorded their final ratings. Between each round, each judge’s individual ratings, including item mean, median, standard deviation and overall passing scores, were provided as feedback to judges before they submitted subsequent ratings. In cases of significant discrepancies after the second round, judges discussed and justified their ratings before making final decisions. Feedback from ratings and discussions aimed to improve consensus [33, 56]. Responses were recorded using Google Forms, with data automatically generated in linked Google Sheets.

For the modified Angoff method, for OBA items, judges estimated the probability that a borderline candidate would correctly answer each question [29, 59]. Angoff ratings ranged from 0 to 100, with lower values indicating greater difficulty and higher values indicating an easier item for borderline candidates. For SAQs, the maximum possible score per item was 10% and judges predicted the score a borderline candidate would achieve [35]. Item-level ratings were calculated by averaging judges’ total ratings for each item. Each judge’s passing score was determined by averaging their total scores across all test items. The final passing score for each test format was obtained by averaging all judges’ individual passing scores. All results were expressed as percentages from 0 to 100.

In the Ebel method, judges classified each OBA and SAQ item into one of nine cells (categories within a 3 × 3 matrix) by making estimations based on difficulty (easy, moderate and difficult) and content relevance (essential, important and additional) [60]. In addition to rating individual items, judges reached a consensus on the percentage of borderline candidates expected to answer correctly within each Ebel grid cell. This percentage was multiplied by the number of items in each cell and the resulting values were summed to calculate the total cell product. Each judge’s passing score was then determined by dividing the total cell product by the total number of test items. The final passing score for each test format was calculated by averaging all judges’ individual passing scores [19].

### Data analysis

Descriptive statistics were used to analyse participants’ demographic data. Differences between judges were examined using the Mann–Whitney U-test and Fisher’s Exact Test, depending on the variable type. Judges were categorised as apprentice (< 5 years) or senior (≥ 5 years) academics, aligning with standard higher education promotion policies. Non-parametric tests were selected owing to the small sample size and non-normal data distribution. Data were analysed using IBM SPSS Version 29 (IBM Corp., Armonk, NY, USA), with statistical significance set at *p* < 0.05.

Each standard setting method comprised three rating rounds, with the final ratings determining the passing scores for this study. Passing scores at the item and judge levels across different methods and test formats were analysed descriptively. Boxplots illustrated the central tendency (median), variability (interquartile range, IQR) and skewness of passing score distributions for OBA and SAQ tests using the modified Angoff and Ebel methods in Groups A and B. Method replicability across different panels was assessed using the Mann–Whitney U-test to compare passing scores between Groups A and B for each method. The Wilcoxon signed-rank test compared passing scores between the modified Angoff and Ebel methods within the groups.

Inter-rater reliability for the modified Angoff method was estimated using the intraclass correlation coefficient (ICC) based on a two-way random-effects model with multiple ratings and absolute agreement. Reliability was classified as follows: ICC < 0.50 = poor, 0.50–0.75 = moderate, 0.76–0.90 = good and 0.91–1.00 = excellent [61]. In the Ebel method, agreement was estimated based on relevancy and difficulty category using Fleiss Kappa calculation [62]. Reliability was interpreted according to the following grading scale: Kappa statistics: < 0.00 = poor, 0.00–0.20 = slight, 0.21–0.40 = fair, 0.41–0.60 = moderate, 0.61–0.80 = substantial and 0.81–1.00 = almost perfect [63].

## Results

Table 1 presents the judges’ demographic characteristics. The median (IQR) years of teaching experience in dentistry for Group A was 14.0 (17.0) and Group B was 21.5 (18.0). Each group consisted of one male and five females, with five participants of Malay ethnicity and one of Chinese ethnicity. Group A has 3 associate professors whereas Group B has 4. Each group was represented by a variation of specialists from different disciplines. No significant differences (*p* < 0.05) were observed between Groups A and B in terms of judge numbers, teaching experience, gender, ethnicity, or academic position as associate professor.

**Table 1:** Demographic characteristics of judges attending standard setting workshop

	Group A	Group B	Total	*p*-value
Total participants (*n*)	6	6	12	
Gender				
Male (*n*,%)	1 (16.7)	1 (16.7)	2(16.7)	1.00^b^
Female (*n*,%)	5 (83.3)	5 (83.3)	10 (83.3)	
Ethnic group				
Malay (*n*,%)	5 (83.3)	5 (83.3)	10 (83.3)	1.00^b^
Chinese (*n*,%)	1 (16.7)	1 (16.7)	2(16.7)	
Experience as teaching staff				
3–9 years (*n*,%)	2 (33.3)	2 (33.3)	4 (33.3)	1.00^b^
> 10 years (*n*,%)	4 (66.7)	4 (66.7)	8 (66.7)	
Mean (SD), years	14.7 (8.7)	17.8 (9.1)	16.3 (8.6)	
Median (IQR), years	14.0 (17.0)	21.5 (18.0)	19.5 (23.0)	.567^a^
Academic position as Associate Professor (*n*,%)	3 (50.0)	4 (66.7)	7 (58.3)	1.00^b^
Field of Disciplines	Dental Public Health Endodontics Periodontics Prosthodontics Restorative Dentistry Oral & Maxillofacial Radiology	Dental Public Health Endodontics Periodontics Prosthodontics Restorative Dentistry Oral & Maxillofacial Surgery		

*SD *standard deviation, *IQR *interquartile range

^a^Mann-Whitney U-test, *p* < 0.05 is considered statistically significant

^b^Fisher’s Exact Test

During the workshop, the judges defined a borderline candidate as ‘just sufficient for safe practice and meeting course learning outcomes’. Table 2 presents the judges’ consensus on the expected percentage of borderline candidates answering correctly in the Ebel grid.

**Table 2:** Item categories and expected percentage (%) of success by a borderline candidate in Ebel grid

Relevance Category	Difficulty Category		
	Easy	Moderate	Difficult
Essential	80	60	40
Important	60	50	30
Additional	25	15	10

**Fig. 1:**
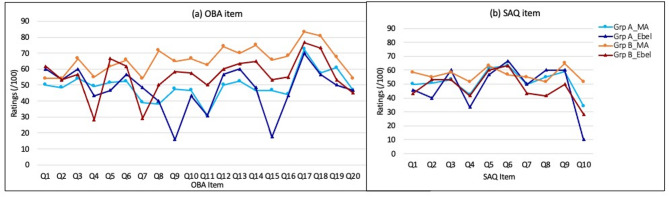
Item-level ratings for the modified Angoff (MA) and Ebel standards for (**a**) OBA item and (**b**) SAQ item

Figure 1 compares item-level ratings for OBA and SAQ test items using the modified Angoff and Ebel methods across Groups A and B. In the OBA test (Fig. 1a), the ratings exhibited a wider spread, indicating greater variability in expert judgments across methods and groups. In contrast, the SAQ test (Fig. 1b) displayed a narrower spread in ratings, reflecting more consistency in expert evaluations. However, certain SAQ items still showed notable discrepancies. Specifically, items 9 and 15 had significant variations in ratings between groups and methods, while item 10 demonstrated the greatest difference among SAQ items.

**Fig. 2:**
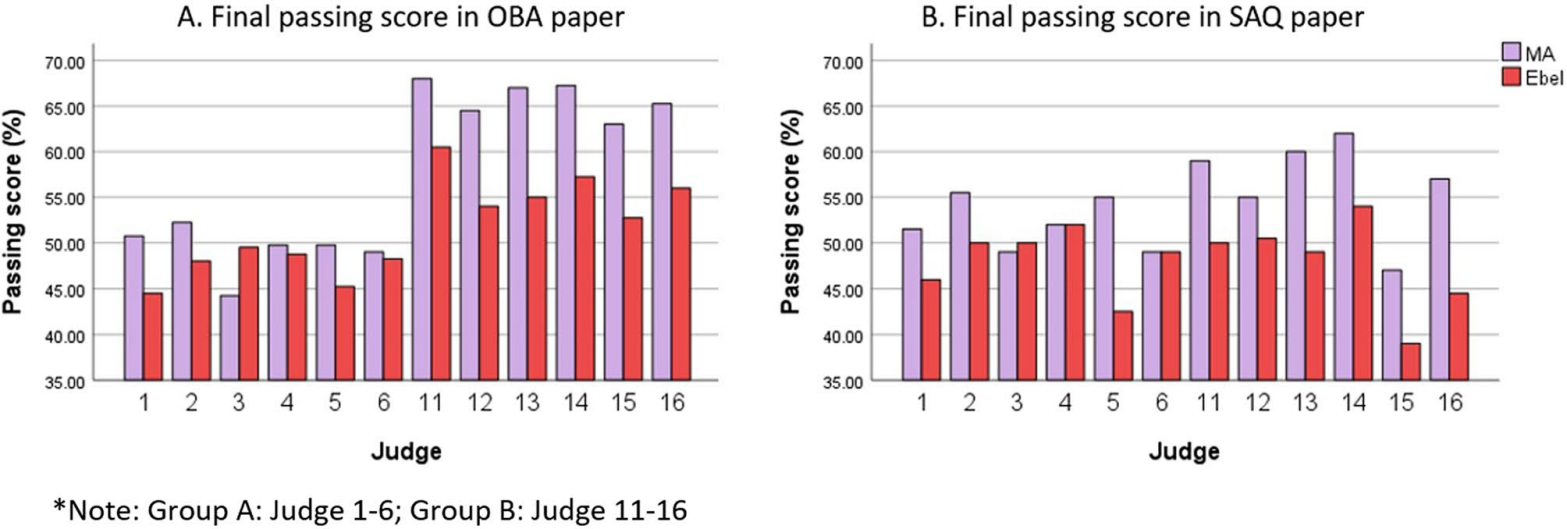
Judges’ final passing score in the theory papers for the modified Angoff (MA) and Ebel standard

**Fig. 3:**
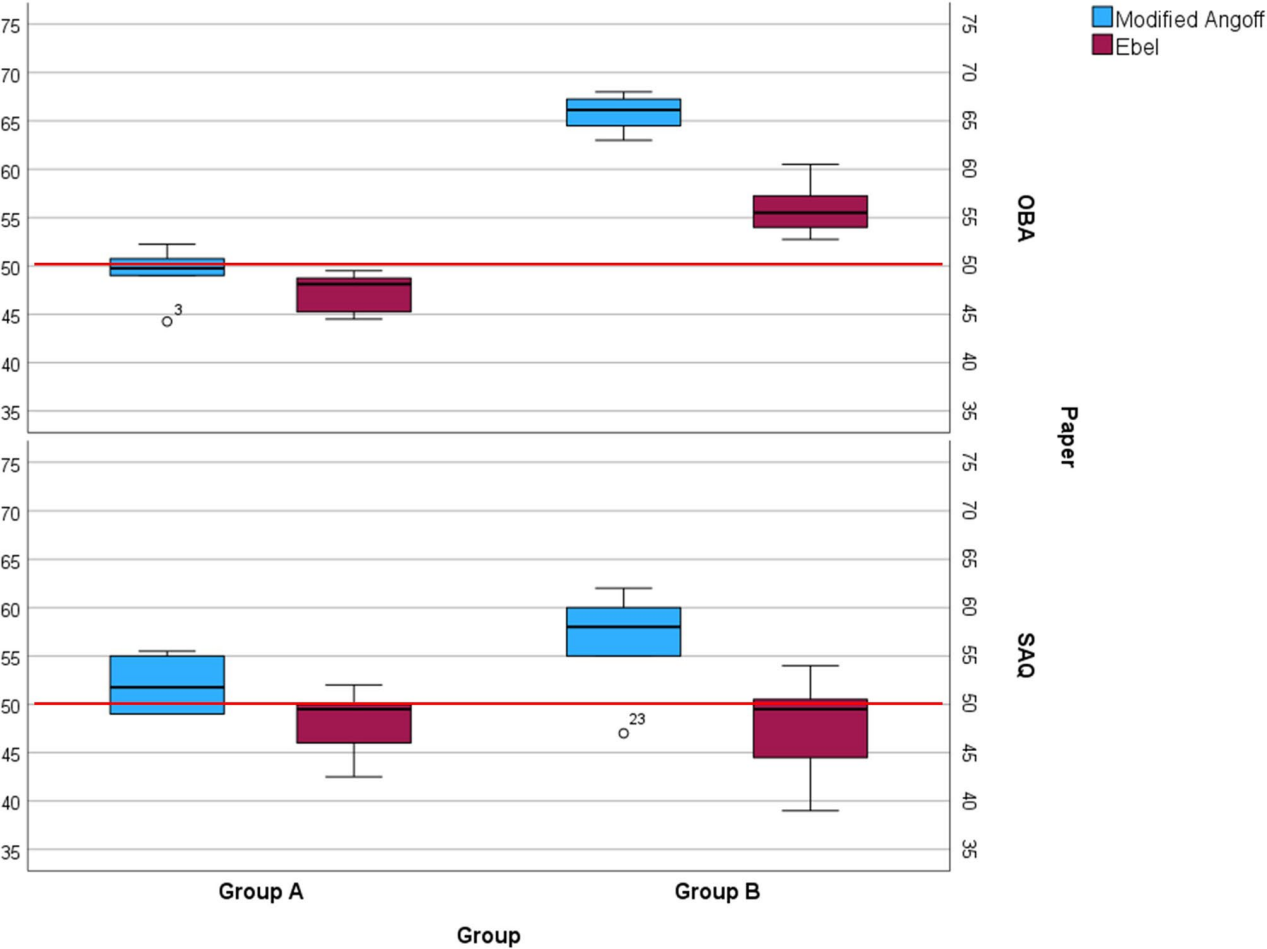
Box plot of passing scores (median) in OBA and SAQ papers when applying MA and Ebel standards

Figure 2 displays individual judges’ passing scores for OBA and SAQ tests across both methods. Most judges assigned higher passing scores using the modified Angoff method than the Ebel method, except for Judge 3. The boxplot in Fig. 3 illustrates the spread of passing scores for OBA and SAQ tests across groups and methods. Note that the red horizontal line at 50% represents the current passing score used at UKM.

Table 3 presents the median (IQR) and mean (SD) passing scores for both test formats. Passing score reproducibility between Groups A and B was assessed using the Mann–Whitney U-test. For the SAQ test, passing scores were consistent across groups for both the modified Angoff method (median, IQR: Group A = 51.75 [6.13]; Group B = 58.00 [7.50]) and the Ebel method (Group A = 49.50 [5.38]; Group B = 49.50 [8.25]). However, for the OBA test, significant differences were observed between groups. Group B assigned higher passing scores than Group A for both methods, with a mean difference of 16.37% in the modified Angoff method (Z = − 2.89, *p* = 0.004) and 7.37% in the Ebel method (Z = − 2.88, *p* = 0.004).

**Table 3:** Median (IQR) and mean (SD) final passing scores (Rating 3) for theory papers in group A and B using modified Angoff and Ebel methods

Test Format	Modified Angoff Method			Ebel Method			
	Groups	Median (IQR)	Mean (SD)	Median (IQR)	Mean (SD)	Z-value^^	*p*-value^^
OBA	A (n=6)	49.75 (3.31)	49.29 (2.71)	48.13 (3.88)	47.38 (2.02)	−1.15	.249
	B (n=6)	66.13 (3.31)	65.83 (1.91)	55.50 (4.38)	55.92 (2.73)	-2.20	.028*
	Z-value^	−2.89		−2.88			
	*p*-value^	.004*		.004*			
SAQ	A (n=6)	51.75 (6.13)	52.00 (2.81)	49.50 (5.38)	48.25 (3.43)	−1.47	.141
	B (n=6)	58.00 (7.50)	56.68 (5.32)	49.50 (8.25)	47.83 (5.30)	-2.21	.027*
	Z-value^	−1.69		-.081			
	*p*-value^	.092		.936			

*IQR* Interquartile Range, *SD* Standard Deviation

(*) indicates a statistical significance (*p* < 0.05)

Mann-Whitney U-test (^)

Wilcoxon Signed Ranks test (^^)

Passing scores determined using the modified Angoff and Ebel methods varied within Groups A and B. The Wilcoxon signed-rank test showed no significant difference in Group A judges’ passing scores between the two methods, regardless of test format. The median difference was 1.62% (Z = − 1.15, *p* = 0.249) for the OBA test and 2.25% (Z = − 1.47, *p* = 0.141) for the SAQ test. However, Group B judges assigned significantly higher passing scores using the modified Angoff method than the Ebel method, with mean differences of 10.62% (Z = − 2.20, *p* = 0.028) for the OBA test and 8.50% (Z = − 2.21, *p* = 0.027) for the SAQ test.

For the inter-rater agreement results, intraclass correlation coefficients (ICC) were rated good in both Groups A (0.870) and B (0.803) in the modified Angoff standard. In the SAQ test, Group A maintained good reliability (ICC = 0.877), whereas Group B was rated as poor (ICC = 0.469) (Table 4). Under the Ebel method, inter-rater agreement was evaluated using Fleiss’ kappa for relevance and difficulty categories (Table 5). In Group A, agreement was almost perfect (κ = 0.805) in OBA test and substantial (κ = 0.763) in SAQ test for relevance category. For the difficulty category, agreement was fair (κ = 0.256) in OBA test and moderate (κ = 0.466) in SAQ test. Group B demonstrated weaker agreement across both test formats, with Fleiss’ kappa values, κ = 0.389 in OBA test and κ = 0.342 in SAQ test for relevance category. For the difficulty category, judges’ agreement were κ = 0.191 in OBA test and κ = 0.032 in SAQ test.

**Table 4:** Inter-rater agreement between groups in modified Angoff method using ICC (2,k)

Test format	Group	Intraclass Correlation	95% CI	*p*-value	Conclusion
OBA	A (*n* = 6)	0.870	0.758–0.941	< 0.001	Good
	B (*n* = 6)	0.803	0.631–0.911	< 0.001	Good
SAQ	A (*n* = 6)	0.877	0.708–0.964	< 0.001	Good
	B (*n* = 6)	0.469	−0.064-0.964	0.039	Poor

ICC value: <0.5 = Poor, 0.50–0.75 = Moderate, 0.76–0.90 = Good, 0.91-1.00 = Excellent

*ICC *Intraclass Correlation Coefficient

**Table 5:** Inter-rater agreement within group A and B using fleiss’ kappa, κ in Ebel method based on relevancy and difficulty category

Test format	Group	Relevancy				Difficulty			
		Kappa	95% CI	*p*-value	Conclusion	Kappa	95% CI	*p*-value	Conclusion
OBA	A (*n* = 6)	0.805	0.717–0.894	< 0.001	Almost perfect	0.256	0.157–0.354	< 0.001	Fair
	B (*n* = 6)	0.389	0.289–0.489	< 0.001	Fair	0.191	0.108–0.273	< 0.001	Slight
SAQ	A (*n* = 6)	0.763	0.640–0.887	< 0.001	Substantial	0.466	0.331–0.601	< 0.001	Moderate
	B (*n* = 6)	0.342	0.202–0.481	< 0.001	Fair	0.032	−0.103-0.168	0.641	Poor

Kappa statistics: <0.00 = Poor, 0.00-0.20 = Slight, 0.21–0.40 = Fair, 0.41–0.60 = Moderate, 0.61–0.80 = Substantial, 0.81-1.00 = almost perfect

## Discussion

This study examined the reproducibility of performance standards by comparing passing scores within and across two groups matched by judges’ characteristics and across two standard setting methods applied to OBA and SAQ tests. Inter-rater agreement was evaluated to support internal validity, while the reproducibility of passing scores across the modified Angoff and Ebel methods addressed external validity.

In the Ebel method, judges first classify items by difficulty and relevance in a grid, then estimate the probability that borderline candidates will answer each category correctly. Variations in defining difficulty and relevance lead institutions to apply different probabilities in the Ebel grid, significantly impacting final scores [60, 62, 64, 65]. In contrast, the modified Angoff method requires judges directly estimate the percentage of borderline candidates expected to answer each item correctly.

Three main factors influence the consistency of ratings during standard setting procedures: judges, test items, and the standard setting process [66]. Panel of judges should represent diverse expertise, experience, stringency, and demographic backgrounds to enhance generalisability [1, 67–69]. Judges should be qualified professionals familiar with the curriculum, learning outcomes, test purpose and students’ expected knowledge levels [70], making academic and clinical lecturers ideal for standard setting practise. This study ensured judge comparability by matching Groups A and B in terms of size, teaching experience, gender, ethnicity and academic rank (associate professor). Group B judges had a higher median level of experience (21.5 years) than Group A (14.0 years), though this difference was not statistically significant (Mann–Whitney U-test, *p* = 0.567). The other difference was the presence of an oral and maxillofacial radiologist in Group A and an oral and maxillofacial surgeon in Group B. However, following standard setting training, all judges were assumed to have adequate content knowledge to determine appropriate undergraduate dental education standards. In multidisciplinary courses such as CDC, exam items cover multiple disciplines. Ideally, panels should include specialists from each discipline. However, assembling ideal number of judges from each of their respective discipline to form a panel of judges within a single institution is impractical. Previous studies suggest that judge specialisation has minimal impact on standard setting outcomes. The findings reported by Hughes (1983) showed greater inconsistency within disciplines than between disciplines, suggesting that the judges’ variation is due to individual differences rather than clinical expertise [47]. Norcini et al. (1988) supported these findings and found that mixed-specialty panels produced comparable passing scores in a critical care medicine certification exam [71]. This study involved eight cardiologists and pulmonologists who also participated in creating and reviewing the items for this certification exam. Although they are experts in narrow specialist fields, their involvement in item development effectively broadened their understanding of the exam content and mitigated heterogeneity in the passing standards. Similarly, in non-medical fields, Plake et al. (1994) found no significant differences in ratings between judges from different subjects (English, mathematics, science and social studies) using the Angoff method [72]. This study had six to seven judges in each content subject to compare 40 expert-content ratings with 10 out-of-content ratings from each of the other three subjects. The small sample size and limited number of items may not be sufficient to detect significant differences using statistical tests. This discrepancy may contribute to greater variation in ratings among judges in Group B. Nevertheless, judges were found to set lower expectations for unfamiliar items and the passing scores increased substantially when these items were removed [73]. Senior judges, particularly specialists who have worked within their expertise for many years, may struggle to align their perceived difficulty of test items with the actual difficulty experienced by examinees in unfamiliar items.

The literature varies on the recommended number of judges for standard setting. Some recommend at least five [21, 74, 75], while others recommend 10 to 15 for MCQ exams using the Angoff method to achieve acceptable standard error [76]. Fowell et al. (2008) advise a minimum of ten judges without discussion, or six with discussion, as the collaborative process can reduce variability in ratings [30]. Ultimately, the ideal number depends on factors like the stakes of the exam, desired standard error, judge variability, and practical constraints [70]. The current study used six judges per panel, following Fowell et al.’s guidelines, though larger panels may improve reliability. All judges were first-time participants and received identical training with detailed written instructions to ensure standardisation.

Our findings showed that the modified Angoff and Ebel standard setting ratings were reproducible across different groups for constructed-response items (SAQ) but not for multiple-choice items (OBA). OBA items in this test consist of four plausible distractors which increase the challenge making the question more challenging to identify the correct option. Judges may therefore find it challenging to predict the performance of minimally competent candidates in selecting the correct answer. In contrast, SAQ items tend to elicit responses that are less influenced by chance, enabling judges to rely more on content mastery and leading to more reproducible standards. Notably, current literature on reproducibility largely focuses on MCQs, with limited research addressing constructed-response items. Previous research on the reproducibility of Angoff standard setting across different panels has demonstrated consistency in passing scores for MCQs in high-stakes examinations [31, 57, 58]. A large-scale study by Tannenbaum and Kannan (2015) examined nine national educator licensure assessments using Angoff method, with two independent panels of 14–23 judges setting standards for examination items in their respective content domains [31]. The authors found minimal differences in item-level judgements and final passing scores across panels, with consistency unaffected by judges’ discussions. Similarly, Norcini and Shea (1992) conducted standard setting for medical examinations with four independent panels and reported consistent Angoff ratings across groups panels, with high correlations among the average item estimates for the total set of items ranging from 0.92 to 0.97 in two sets of examinations [58]. Comparable findings in finance management assessments further support the reliability of Angoff-based ratings [57]. The consistency observed in these studies may be attributed to judges having access to item *p*-values (item difficulties), which aid their evaluations [57, 58]. However, studies on the reproducibility of the Ebel method across different panels are scarce.

Our study found varying levels of consistency between the modified Angoff and Ebel methods across groups. Limited recent evidence directly compares these methods in healthcare professional assessments. Hughes (1983) reported that the Angoff and Ebel ratings were consistent, differing by less than 1% [47]. When judges applied these methods without access to candidates’ performance data, our findings aligned with existing literature [48, 52]. We observed that the modified Angoff method generally produced higher passing scores than the Ebel method. However, this difference was statistically significant only in Group B, where the modified Angoff method resulted in higher scores. In Group A, the modified Angoff method yielded scores that were 1.6% and 2.3% higher Ebel ratings for the OBA and SAQ tests, respectively. Yet, this difference was not statistically significant. A larger number of judges is often needed to detect statistically significant differences when the effect size is small, and the practical difference of 1–2% should be taken into account.

Although this study did not aim to compare passing scores determined by the modified Angoff and Ebel methods with the faculty’s fixed 50% standard, it is notable that Group A’s passing scores were generally aligned with this benchmark across all standard setting methods and test formats. In contrast, Group B’s ratings deviated more substantially (Fig. 3).

Despite using the same test items, Group A exhibited greater agreement between the two methods than Group B. Modified Angoff ratings also demonstrated higher inter-rater agreement than Ebel ratings, consistent with prior studies. Reported inter-rater agreement for the modified Angoff method with percentage method ranged from 0.81 to 0.82 [77], while the modified Angoff method with Yes/No/Maybe method ranged from 0.65 to 0.82 [43]. In contrast, Ebel ratings tend to fall below 0.15 for both difficulty and relevance categories [62]. However, these inter-rater agreement values were based on individual ratings with no discussion and performance data. The variation among judges, measured by root mean square error was shown to decrease after the discussion round alone [30]. The consistency among the judges has implications for the internal validity evidence of the suggested standards and it indirectly strengthens the integrity of the procedures [20, 43]. Poor consistency among the judges may reflect their different understandings of the performance standard for defining minimum competency, judges’ cognitive abilities to conceptualise minimal pass level for borderline students and judges are unclear about the standard setting procedure [78–80].

To improve internal consistency, previous studies recommend providing candidate performance data to enhance intra-rater reliability [49–51]. Introducing p-value, examinees’ cumulative scores distribution, and discussion improved judges’ rating reliability and increased alignment with item difficulty [50]. Although three of seven tests showed small but significant changes in cut scores, the absence of a control group made it difficult to isolate the effects of discussion or performance data. To address this, Novakovic (2008) compared judges’ ratings after discussion alone to their ratings following both discussion and performance data. While item-level correlations improved with the addition of difficulty and discrimination indices, changes in final passing scores were negligible [51]. Judges in the present study lacked access to performance data. Since all judges were new to standard setting, introducing additional data could have increased the cognitive load, affecting their learning experience and influencing decisions. Research shows judges rely heavily on performance data when presented, often responding mechanically rather than making content-based decisions [81]. Clauser et al. (2009) found that initial judgments made without data, showed weak correlation with empirical item difficulties. After reviewing accurate or manipulated data, judges significantly altered their decisions, even when the data were artificially manipulated. While performance data can enhance reliability, their influence on final scores and the risk of judges deferring to data over expertise must be critically considered.

The current research compares the modified Angoff and Ebel method in multidisciplinary summative knowledge assessments for the final professional examination. However, these methods can also be applied to individual course within the dental curriculum at various stakes level [6, 45, 82–84]. Panels are required to establish a working definition of the ‘borderline student’ for progress testing or certification examinations to ensure that the standards set are aligned with the expected levels of performance.

Civek (2020) described that validity is a collection of evidence quality and the degree to which that evidence supports the intended meaning of the scores [46]. The authors believe that validity should not be treated as a dichotomised entity. Additional evidence, such as intra-rater consistency in relation to empirical item difficulties, decision consistency, and comparisons with other external sources of information, could further strengthen both the internal and external validity of the passing scores.

### Limitations and future recommendations

This study has several limitations that should be considered. First, this study only involved judges from a single dental institution in Malaysia, limiting its generalisability. Future research should expand standard setting practices to dental assessments at a national level, involving a diverse panel of judges from multiple institutions across Malaysia. However, this is the first study in dental education to compare the reproducibility of the modified Angoff and Ebel methods across two matched panels, providing valuable insights into passing scores for OBA and SAQ tests.

Second, although statistical tests showed no significant difference in experience levels between Group A (median = 14.0 years) and Group B (median = 21.5 years), the 7.5-year gap may have influenced the judges’ decisions. Previous research has shown that judges with higher academic qualifications and expertise are associated with greater variability and higher passing scores [83, 85]. Therefore, the results should be interpreted with careful consideration of the panel’s composition.

Third, the number of test items included in this standard setting exercise was relatively small, raising concerns about the reliability of the results. A simulation study by Shulruf et al. (2018) suggested that a combination of 80 items is required if 15 judges are used, or 15 items are required if 20 judges are used in a single-round Angoff standard setting procedure [68]. Increasing the number of items may reduce the standard error of measurement in passing score estimations. However, this was not feasible in the present study due to several constraints. For the CDC course, the total number of items in the MCQ test was 20, while the SAQ test contained only 10 items. Additionally, all judges were new to the standard setting process. Concerns about cognitive overload, potential for fatigue effects and time constraints further limited the number of items that could be included.

Finally, this study employed the classical test theory to examine the reproducibility of different standard setting methods and reliability among the judges. Future research could incorporate alternative statistical model, such as Generalisability Theory (G-Theory), to provide further reliability and validity evidence. G-theory can help identify sources of variance attributable to judges or items or interaction of both facets in cut-score judgements [39, 86–89]. Consequently, appropriate measures, such as judge selection, training or item refinement, can be implemented to improve future practice.

## Conclusion

This study provides insights into the reproducibility of standard setting methods across panels and different test formats in dental assessment and consistency among judges when making judgements using the modified Angoff and Ebel methods. Reproducibility of modified Angoff and Ebel standards across panels was mixed. Within this study’s limitations, we conclude that the passing scores were consistent across panels for the modified Angoff and Ebel method for the SAQ test but not in the OBA test. In the OBA test, Group B rated higher passing standards than Group A in both methods. Within each group, passing scores set by Group A did not considerably differ between the modified Angoff and Ebel methods in both the OBA and SAQ tests. Conversely, Group B rated higher passing scores in the modified Angoff method than in the Ebel method in both OBA and SAQ tests.

Additionally, the inter-rater agreement in Group A was better than that in Group B in both methods. The modified Angoff method demonstrated generally better agreement ratings than the Ebel method. The reproducibility of the passing scores between the groups and strong inter-rater agreements highlight important information regarding the consistency of these methods and judges’ decision-making processes. Policymakers should carefully consider these findings when making pass-fail decisions for establishing defensible and reliable passing standards instead of random decisions from panels.

## Supplementary Information

Supplementary Material 1.

## Data Availability

The datasets collected and/or analysed during the current study are not publicly available but are available from the first author, Ting Khee Ho on request.

## Abbreviations

CDC: Comprehensive Dental Care
MCQ: multiple-choice question
SAQ: short answer questions
OBA: one-best answer
