# Supplementary material for: Standard setting for dental knowledge tests: reproducibility of the modified Angoff and Ebel method across judges
Source: BMC Med Educ. 2025 Oct 15;25:1426. doi: 10.1186/s12909-025-07822-3 (PMC12522590; doi:10.1186/s12909-025-07822-3)
Supplement: Supplementary file 1 — Supplementary Material 1. [file 12909_2025_7822_MOESM1_ESM.docx]

Detailed timetable for the standard setting workshop

| **Title** | **Objective** | **Method** | **Duration** |
| --- | --- | --- | --- |
| **Day 1** |  |  |  |
| Introduction to Standard Setting | - Introduction of education standards and overview on standard setting including definitions, types of cut scores, classification, applications, validity criteria of education measurement. | Lecture | 1 hour |
| A Dive from Standard to Competencies | - Overview on intended learning outcomes for dental programme and concept of constructive alignment. | Lecture | 30 mins |
| Criterion-referenced Standard Setting: Test-centred Standard and Standard Setting Protocol | - Brief on Competencies of New Dental Graduates in Malaysia and performance standard. - Explaining concept and calculation of Angoff and modified Angoff, Ebel and Nedelsky standard. - Brief on essential steps in setting standard for test-centred standard | Lecture | 2 hours |
| Criterion-referenced Standard Setting: Examinee-centred Standard | - Explaining concept and calculation of Contrasting group, borderline group and borderline regression standard. | Lecture | 1 hour |
| Item analysis on performance data | - Explaining the item analysis for OBA items which includes interpretation of item difficulty index, item discriminative index and non-functioning distractor. | Lecture | 1 hour |
| Definition of borderline candidate | - Discuss concept on borderline candidate and reaching an understanding and definition for Borderline candidate. | Group discussion | 30 mins |
| Practical sessions to standard set exam items using Modified Angoff and Ebel method. | - Practise in setting standard for 10 OBA and 5 SAQ items by applying Modified Angoff and Ebel method. | Group work and discussion | 2 hours |
| **Day 2** |  |  |  |
| Standard setting | - Briefing on the standard setting procedure and course learning outcomes. | Lecture | 15 mins |
| Modified Angoff Round 1 rating | - Standard set the first round of rating. | Individual ratings | 1 hour |
| Feedback | - Reviewing feedback presented by moderator. | Individual reviewing | 15 mins |
| Modified Angoff Round 2 rating | - Standard set the second round of rating. | Individual ratings | 1 hour |
| Feedback and discussion | - Reviewing feedback presented by moderator. | Group discussion | 15 mins |
| Modified Angoff Round 3 rating | - Standard set final round of rating. | Individual ratings | 1 hour |
| Passing score analysis | - Descriptive analysis of passing score across three ratings. | Group discussion | 15 mins |
| Ebel Round 1 rating | - Standard set the first round of rating. | Individual ratings | 1 hour |
| Feedback | - Reviewing feedback presented by moderator. | Individual reviewing | 15 mins |
| Ebel Round 2 rating | - Standard set the second round of rating. | Individual ratings | 1 hour |
| Feedback and Discussion | - Reviewing feedback presented by moderator. | Group discussion | 15 mins |
| Ebel Round 3 rating | - Standard set final round of rating. | Individual ratings | 1 hour |
| Passing score analysis | - Descriptive analysis of passing score across three ratings | Group discussion | 15 mins |
| **Day 3** |  |  |  |
| Modified Angoff standard for Multiple Performance level (MPL) | - Briefing on the standard setting procedure for MPL and performance level description | Lecture | 30 mins |
| Modified Angoff MPL Round 1 rating | - Standard set the first round of rating. | Individual ratings | 1 hour |
| Feedback | - Reviewing feedback presented by moderator. | Individual reviewing | 15 mins |
| Modified Angoff MPL Round 2 rating | - Standard set the second round of rating. | Individual ratings | 1 hour |
| Feedback and discussion | - Reviewing feedback presented by moderator. | Group discussion | 30 mins |
| Modified Angoff MPL Round 3 rating | - Standard set final round of rating. | Individual ratings | 1 hour |
| Discussion and feedback | - Presenting summary of passing scores of all methods between the two groups and final discussion. | Group discussion | 30 mins |
| Final questionnaire form | - Judges answered questionnaire forms |  | 30 mins |
| Focus group discussion | - Focus group discussion aim to explore the opinion, experiences, and preferences of participants on using different methods of standard setting when determining the pass marks. | Group discussion | I hour |
| Note:  The Angoff and Ebel ratings discussed in this manuscript were collected on Day 2. | | | |
